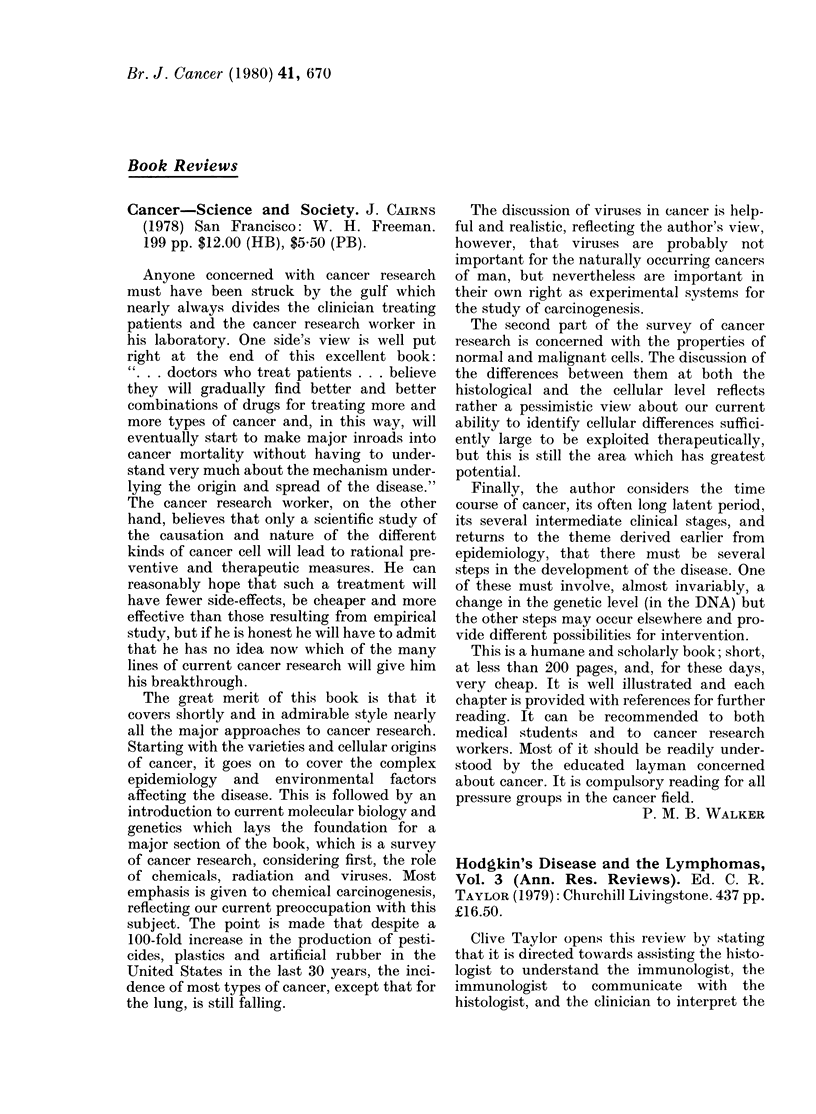# Cancer—Science and Society

**Published:** 1980-04

**Authors:** P. M. B. Walker


*Br. J. Cancer* (1980) **41**, 670

### *Book Reviews*

**Cancer—Science and Society.** J. CAIRNS (1978) San Francisco: W. H. Freeman. 199 pp. $12.00 (HB), $5·50 (PB).

Anyone concerned with cancer research must have been struck by the gulf which nearly always divides the clinician treating patients and the cancer research worker in his laboratory. One side's view is well put right at the end of this excellent book: ". . . doctors who treat patients . . . believe they will gradually find better and better combinations of drugs for treating more and more types of cancer and, in this way, will eventually start to make major inroads into cancer mortality without having to understand very much about the mechanism underlying the origin and spread of the disease." The cancer research worker, on the other hand, believes that only a scientific study of the causation and nature of the different kinds of cancer cell will lead to rational preventive and therapeutic measures. He can reasonably hope that such a treatment will have fewer side-effects, be cheaper and more effective than those resulting from empirical study, but if he is honest he will have to admit that he has no idea now which of the many lines of current cancer research will give him his breakthrough.

The great merit of this book is that it covers shortly and in admirable style nearly all the major approaches to cancer research. Starting with the varieties and cellular origins of cancer, it goes on to cover the complex epidemiology and environmental factors affecting the disease. This is followed by an introduction to current molecular biology and genetics which lays the foundation for a major section of the book, which is a survey of cancer research, considering first, the role of chemicals, radiation and viruses. Most emphasis is given to chemical carcinogenesis, reflecting our current preoccupation with this subject. The point is made that despite a 100-fold increase in the production of pesticides, plastics and artificial rubber in the United States in the last 30 years, the incidence of most types of cancer, except that for the lung, is still falling.

The discussion of viruses in cancer is helpful and realistic, reflecting the author's view, however, that viruses are probably not important for the naturally occurring cancers of man, but nevertheless are important in their own right as experimental systems for the study of carcinogenesis.

The second part of the survey of cancer research is concerned with the properties of normal and malignant cells. The discussion of the differences between them at both the histological and the cellular level reflects rather a pessimistic view about our current ability to identify cellular differences sufficiently large to be exploited therapeutically, but this is still the area which has greatest potential.

Finally, the author considers the time course of cancer, its often long latent period, its several intermediate clinical stages, and returns to the theme derived earlier from epidemiology, that there must be several steps in the development of the disease. One of these must involve, almost invariably, a change in the genetic level (in the DNA) but the other steps may occur elsewhere and provide different possibilities for intervention.

This is a humane and scholarly book; short, at less than 200 pages, and, for these days, very cheap. It is well illustrated and each chapter is provided with references for further reading. It can be recommended to both medical students and to cancer research workers. Most of it should be readily understood by the educated layman concerned about cancer. It is compulsory reading for all pressure groups in the cancer field.

P. M. B. WALKER